# Metastatic seminoma treated with either single agent carboplatin or cisplatin-based combination chemotherapy: a pooled analysis of two randomised trials

**DOI:** 10.1038/sj.bjc.6602020

**Published:** 2004-07-13

**Authors:** C Bokemeyer, C Kollmannsberger, S Stenning, J T Hartmann, A Horwich, C Clemm, A Gerl, C Meisner, C-P Rückerl, H-J Schmoll, L Kanz, T Oliver

**Affiliations:** 1 Department of Hematology/Oncology, University of Tuebingen, Otfried-Mueller-Str. 10, 72076 Tuebingen, Germany; 2 Division of Medical Oncology, British Columbia Cancer Agency, Vancouver, Canada; 3 MRC Trials Office, Medical Research Council, Cambridge, England; 4 Radiotherapy Unit, Royal Marsden Hospital, Surrey, England; 5 Department of Hematology/Oncology, University of Munich, Munich, Germany; 6 Onkologische Praxis, Munich, Germany; 7 Institute for Medical Information Processing, University of Tuebingen, Tuebingen, Germany; 8 Department of Hematology/Oncology, University of Halle, Germany; 9 Department of Medical Oncology, St Barts & The London Hospitals NHS Trust, London, England

**Keywords:** seminoma, carboplatin, cisplatin, metastatic

## Abstract

To study the role of single agent carboplatin chemotherapy in patients with metastatic seminoma based on the data from two randomised trials. In subgroup analyses in patients with different disease characteristics, the outcome treated with either single agent carboplatin or cisplatin-based combination chemotherapy was compared. Individual patient data from two randomised European trials involving patients with metastatic seminoma were gathered. The primary endpoint for all analyses was progression-free survival. The source data of 361 patients, 184 treated with cisplatin-based combinations and 177 treated with carboplatin single agent therapy, were entered into the analysis. Patient characteristics were comparable among the cisplatin-based and the carboplatin single agent treated patient groups with lymph nodes and lungs being the most frequent metastatic sites in 92 and 8% of patients, respectively. Overall, patients treated with single agent carboplatin had an inferior 5-year overall (89 and 94%; *P*=0.09) and progression-free survival rate (72 and 92%; *P*< 0.0001) compared with patients receiving cisplatin-based combinations. For all investigated subgroups (based on age, prior radiation therapy, metastatic sites), carboplatin single agent therapy was found to be inferior to cisplatin-based combination chemotherapy. In conclusion, carboplatin single agent therapy cannot be recommended as standard treatment for any patient subgroup with advanced metastatic seminoma and cisplatin-based combination regimens remain the standard of care.

Today, approximately 70–80% of patient with testicular metastatic germ cell cancer can be cured with standard-dose cisplatin-based combination chemotherapy (Einhorn, 1990; Bosl and Motzer, 1997). With long-term cure rates of 80–95%, patients with metastatic seminoma seem to exhibit an even better prognosis than patients with nonseminomatous germ cell cancer (Mead and for the International Germ Cell Cancer Collaborative Group, 1997). This is also reflected by the fact that the International Germ Cell Cancer Cooperative Group (IGCCCG) classification does not categorise patients with pure seminoma into the ‘poor prognosis’ group (Mead and for the International Germ Cell Cancer Collaborative Group, 1997). Three and four cycles of bleomycin, etoposide, and cisplatin (BEP) are considered the standard treatment for patients with metastatic seminoma within the good and intermediate prognosis criteria IGCCCG group, respectively (Mead and for the International Germ Cell Cancer Collaborative Group, 1997). However, since most patients with seminoma are diagnosed at stage I and widespread metastatic disease is rare, only a few studies regarding the optimal treatment of patients with metastatic seminoma have been performed. Due to the exceptionally high cure rate of patients with advanced seminoma, the reduction of treatment-related toxicity, while at the same time maintaining efficacy, has been the focus of investigations in the past years. Cisplatin-based combination chemotherapy was reported to cause significant acute and long-term side effects in the population of seminoma patients, who are usually about 10–15 years older than patients with nonseminomatous disease. Since cisplatin was reported to have a high single agent activity in seminoma and due to the availability of the cisplatin analogue carboplatin with an improved toxicity profile as compared to its parent compound, phase II studies have investigated single agent carboplatin therapy in patients with metastatic seminoma (Oliver *et al*, 1990; Horwich *et al*, 1992; Schmoll *et al*, 1993). Horwich *et al* (1992) reported an excellent 3-year overall survival rate of 91% for single agent carboplatin in 70 patients with metastatic seminoma. Similar results were reported by Schmoll *et al* (1993) and Oliver *et al* (1990). Therapy-associated toxicities were very moderate in all three studies.

These results led to the initiation of two randomised phase III trials, one in Great Britain and one in Germany comparing carboplatin single agent therapy with cisplatin-based combinations. In the British trial single agent carboplatin was compared with the combination of cisplatin and etoposide whereas in the German study the combination of cisplatin, etoposide and ifosfamide was chosen as the standard arm. The British seminoma study was closed early after the enrollment of 130 patients due to poor accrual and to results of randomised trials demonstrating inferiority of carboplatin-based combinations compared with cisplatin-based regimens in patients with metastatic nonseminoma (Bokemeyer *et al*, 1996; Horwich *et al*, 2000). The final results of the German randomised seminoma trial on single agent carboplatin have not yet been fully published; however, the first analysis after completion of accrual has indicated an inferiority of carboplatin single agent therapy regarding progression-free survival (Clemm *et al*, 2000). Neither the British nor the German study had included enough patients to answer two important questions in advanced seminoma patients: First, are there any subgroups among patients with metastatic seminoma, which may still achieve an equally good outcome with carboplatin single agent therapy as with cisplatin-based combinations? Second, which pretreatment factors predict the chances of survival after chemotherapy for metastatic seminoma? We have therefore combined the individual patient data from both European randomised trials in this analysis in order to search for possible patient subgroups with equivalent outcome after either carboplatin single agent therapy or cisplatin combination therapy.

## PATIENTS AND METHODS

The individual patient data of 130 patients with advanced seminoma included in the British trial and of 250 patients included in the German randomised phase III trial were obtained (Clemm *et al*, 2000; Horwich *et al*, 2000). In total, 19 patients were excluded from the analysis due to missing data. All patients had been treated with either four to six cycles of carboplatin single agent therapy or four cycles of cisplatin-based combination chemotherapy either at initial diagnosis or at relapse after radiotherapy for stages I or IIA/B disease. Within the German randomised trial, patients received four to six cycles of carboplatin at a dose of 400 mg m^−2^ (actual dose corrected according to creatinine clearance) given on day 1 of a 4-week cycle or 4 cycles of the VIP regimen consisting of etoposide 100 mg m^−2^, ifosfamide 1200 mg m^−2^ and cisplatin 20 mg m^−2^, all administered days 1 through 5 of a 4-week cycle. This study recruited patients from July 1990 through August 1999. The VIP regimen was chosen as the standard arm in the German randomised trial since VIP had shown a high complete remission rate, no pulmonary toxicity and a very low relapse rate in a preceding phase II trial in patients with seminoma (Clemm *et al*, 1993).

Patients in the British randomised study were treated with four cycles of carboplatin 400 mg m^−2^ (actual dose corrected according to creatinine clearance) given on day 1 or with the combination of etoposide 120 mg m^−2^ days 1–3 and cisplatin 20 mg m^−2^ days 1–5. Both regimens were given in 3-week intervals. The British trial started in 1990 and was closed early after 130 patients had been randomised following the recommendation of the independent data monitoring committee. Inclusion criteria were almost identical for both trials. Advanced disease was defined as the presence of abdominal lymph node metastases >5 cm, supradiaphragmatic lymph nodes or visceral metastases. High-dose chemotherapy with peripheral stem cell support was not used as first-line therapy in any of these patients.

The individual patient data were pooled and included in a SAS data bank (version 8.8; SAS Institute Inc., Cary, NC, USA). A total of 19 patients in whom only an incomplete data set with more than two missing variables was obtained were excluded from further analysis.

Progression-free and overall survival were calculated from the date of randomisation until the date of relapse and the date of death, respectively. Patients were analysed according to the ‘intent-to-treat’ principle.

The primary end point for subgroup analyses was progression-free survival, which was calculated from the date of randomisation to the date of disease progression. Survival rates as well as median follow-up were calculated using the Kaplan–Meier method. Comparisons in survival were carried out using the Log-Rank test (Kaplan and Meier, 1958; Cox and Oakes, 1984). The following subgroups were investigated: patients with lung metastases (*n*=29); without lung metastases (*n*=332); with lymph node metastases only (*n*=263); without lymph node metastases (*n*=98); with nonpulmonary visceral metastases (*n*=20); without nonpulmonary visceral metastases (*n*=341); patients with relapse after prior radiation for stage I and IIA/B disease (*n*=51); without prior radiation (*n*=310); patients younger than 30 years of age (*n*=59); patients between 30 and 50 years of age (*n*=221); patients older than 50 years of age (*n*=56) (age missing in 25 patients).

Data are presented with two-tailed *P*-values (unadjusted for multiple comparisons) and 95% confidence intervals for the hazard ratios. Computations were performed using SAS-PC for windows (version 8.0; SAS Institute Inc., Cary, NC, USA).

## RESULTS

A total of 361 patients with complete data from both randomised trials were entered into the pooled analysis.

In the German trial a progression-free and overall survival rate of 72% [95% CI: 63–80%] and 90% [95% CI: 85–96%], respectively, for the carboplatin treated patients and of 95% [95% CI: 91–99%] and 95% [95% CI: 91–98%], respectively, for the cisplatin group was reported after a median follow-up of 52 months [2–112 months]. Similar results have been achieved by the British study with a progression-free and overall survival rate of 72% [95% CI: 60–83%] and 87% [95% CI: 78–96%] for the carboplatin patients and of 85% [95% CI: 76–94%] and 91% [95% CI: 83–98%] for the patients receiving cisplatin-based combinations. Median follow-up in the British trial was 54 [2–112] months.

After pooling the individual patient data from both trials, a total of 177 (49%) patients were treated with carboplatin single agent therapy and 184 (51%) patients with cisplatin-based combinations. Patient characteristics were well balanced between both groups (Table 1

**Table 1:** Patient characteristics and treatment results

	**Carboplatin (*n*=177)**	**Cisplatin (*n*=184)**	**Total (*n*=361)**
Median age (years)	37 [15–64]	39 [21–71]	38 [15–71]
Gonadal/extragonadal	86%/14%	83%/17%	84%/16%
*Prior radiation therapy*			
No	85%	84%	84%
Yes	14%	14%	14%
No data	1%	2%	2%
			
*Metastatic sites*			
Lymph nodes	91%	92%	92%
Nonpul. visceral	6%	5%	6%
Pulmonary	6%	10%	8%
			
*IGCCCG stage at diagnosis*			
Good	94%	95%	94%
Intermediate	6%	5%	6%
			
*Response to treatment*			
CR/PR	91%	95%	93%
SD/PD	9%	5%	7%
			
*Relapse*			
Yes	29%	9%	19%
			
*Salvage therapy*	*n*=52	*n*=17	
SD-CTx	77%	59%	72%
HD-CTx	0%	6%	1%
Radiation	10%	24%	13%
No treatment	2%	0%	1%
No data	12%	12%	12%
			
Median follow-up (years)	4.5 [0,3–9]	4.5 [0.2–9]	4.5 [0.2–9]

SD-CTx=standard-dose chemotherapy; HD-CTx=high-dose chemotherapy; CR=complete remission; PR=partial remission; SD=stable disease; PD=progressive disease.

). Of these 361 patients, 14% had relapsed after previous radiation therapy. Median ages were 37 [range: 15–64 years] and 39 years [range: 21–71 years] for patients treated with carboplatin and cisplatin-based therapy, respectively. Most patients had gonadal primary tumours. Nonpulmonary visceral metastases were present in 10 patients of each group, pulmonary metastases in 11 patients of the carboplatin group, and 18 patients of the cisplatin group. Lymph node metastases were the most common metastatic sites in both groups (161 and 169 patients in the carboplatin and cisplatin-based treated group, respectively).

In total, 90% of all patients belonged to the IGCCCG ‘good prognosis’ group and 10% to the ‘intermediate prognosis’ group. The median follow-up for all 361 patients was 4.5 years (range: 0.2–9 years) being virtually identical for both patient groups (4.6 years for the carboplatin group and 4.5 years for the cisplatin group).

Haematological as well as non-haematological side effects were significantly more frequent in the cisplatin-based combination group than in the carboplatin treated patients (Table 2

**Table 2:** Maximum toxicity of carboplatin single agent therapy as compared to VIP or EP

		**British trial**		**German trial**	
	**WHO**	**Carboplatin (%)**	**EP (%)**	**Carboplatin (%)**	**VIP (%)**
*Maximum haematological toxicity*					
Thrombocytopaenia	Grade 1/2	35	6	12	14
	Grade 3/4	9	8	6	27
Neutropaenia	Grade 1/2	69	63	34	14
	Grade 3/4	4	32	6	68
					
*Maximum nonhaematological toxicity*					
Nausea/vomiting	Grade 3/4	9	15	5	20
Diarrhoea	Grade ⩾ 1	5	15	12	19
Neurotoxicity	Grade ⩾ 1	0	11	2	14
Ototoxicity	Grade ⩾ 1	0	15	3	3

). Neither neurotoxicity nor ototoxicity exceeded WHO grade 2.

**Figure 1:**
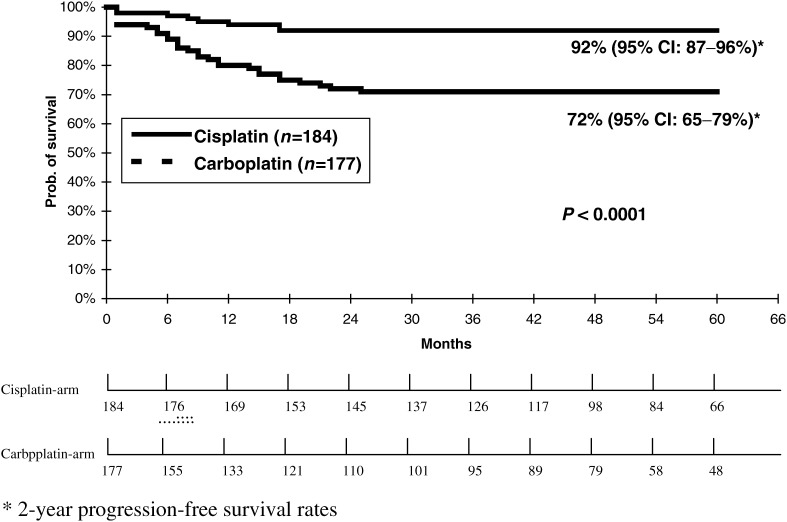
Progression-free survival rates for patients from two randomised studies receiving either cisplatin-based combination chemotherapy or carboplatin single agent therapy for metastatic seminoma (*n*=361).

**Figure 2:**
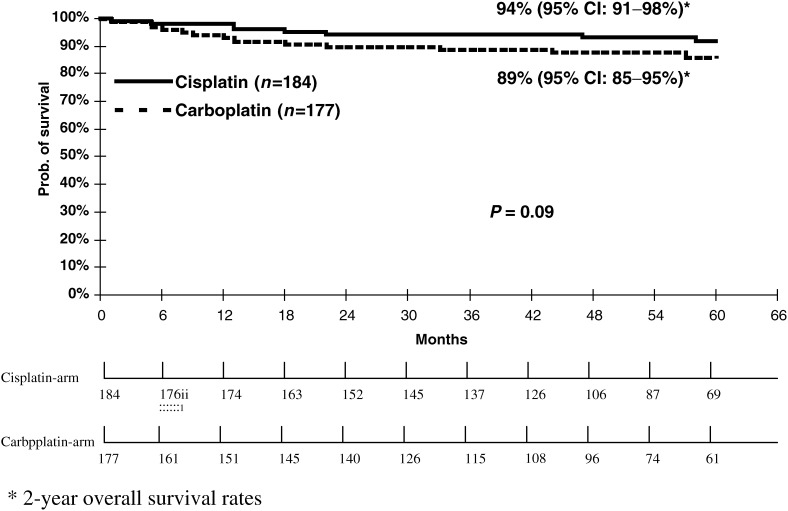
Overall survival for patients from two randomised studies receiving either cisplatin-based combination chemotherapy or carboplatin single agent therapy for metastatic seminoma (*n*=361).

Relapses occurred in 52 (29% [95% CI: 23–36%]) of the carboplatin treated patients whereas only 17 (9% [95% CI: 5–13%]) relapses were observed following treatment with cisplatin-based regimens (*P*<0.05). This resulted in an inferior 2-year progression-free survival rate for carboplatin single agent therapy as compared to cisplatin-based combinations with 72% [95% CI: 65–79%] *vs* 92% [95% CI: 87–96 %] (*P*< 0.001) (Figure 1

). After a median follow-up of approximately 4.5 years for both groups, 36 patients had died, 22 (12% [95% CI: 7-17%]) in the carboplatin, and 14 (8% [95% CI: 4–12%]) in the cisplatin group, which resulted in a nonsignificant difference of 5% in overall survival in favour of cisplatin-based combinations (2-year overall survival 90% [95% CI: 85–95%] for carboplatin treated patients *vs* 94% [95% CI: 91–98 %] for patients receiving cisplatin-based combinations; *P*=0.09) (Figure 2

). Four patients died during treatment with cisplatin-based combination chemotherapy, three in the VIP group, and one in the EP group, whereas only one patient died from carboplatin treatment.

For all the investigated subgroups the progression-free survival rates achieved with cisplatin-based combination therapy were superior to those following treatment with carboplatin single agent therapy. Table 3

**Table 3:** Subgroup analyses for patients from two randomised studies (*n*=361): The hazard ratio gives the relative increase of the risk for disease progression following carboplatin therapy compared to cisplatin-based combination treatment. Inferiority of carboplatin has been observed in all investigated subgroups

**Subgroup**	***N* pts**	**Hazard ratio for PFS [95% CI]**
Lung metastases	29	1.4 [0.4–5.2]
No lung metastases	332	4.6 [2.5–8.7]
Lymph node metastases only	263	3.7 [1.9–7.3]
No lymph node metastases	98	3.6 [1.4–9.5]
Nonpulmonary visceral metastases	20	4.7 [0.5–49.5]
No nonpulmonary visceral metastases	341	3.5 [2.0–6.3]
Age <30 years^a^	59	2.7 [0.5–14.2]
Age 30–50 years^a^	221	2.8 [1.5–5.3]
Age >50 years^a^	56	12.9 [1.7–100.4]
Previous radiation (Yes)	310	2.8 [0.9–9.0]
Previous radiation (No)	51	3.7 [2.0–6.9]

PFS=progression-free survival.

a Age missing in 25 patients.

shows the relative increase in risk expressed as hazard ratios for progression-free survival for patients receiving carboplatin single agent therapy compared to cisplatin-based combinations.

## DISCUSSION

Due to the favourable toxicity profile, carboplatin single agent therapy as well as carboplatin-based combination regimens have been investigated in germ cell cancer patients within a number of phase II and phase III trials (Bajorin *et al*, 1993; Schmoll *et al*, 1993; Bokemeyer *et al*, 1996; Horwich *et al*, 1997). The promising results observed in phase II studies for single agent carboplatin therapy in patients with metastatic seminoma have served as the rationale for two randomised phase III trials conducted in Great Britain and Germany, which compared single agent carboplatin therapy with cisplatin-based combination regimens (Clemm *et al*, 2000; Horwich *et al*, 2000). The British trial, however, was prematurely closed due poor accrual and to reports of inferiority of carboplatin-based therapy in patients with metastatic nonseminoma. The analysis of the 130 seminoma patients randomised until closure of the trial revealed a nonsignificant 10% difference in progression-free and a nonsignificant 5% difference in overall survival in favour of the combination of cisplatin/etoposide (Horwich *et al*, 2000). The results of the German randomised trial based on 250 patients have demonstrated a statistically significant 21% absolute lower progression-free survival rate following carboplatin therapy compared with the combination of cisplatin, etoposide, and ifosfamide (Clemm *et al*, 2000). The overall survival rate achieved with carboplatin single agent therapy was 8% lower than that achieved with cisplatin-based regimens, but this was not of statistically significant difference. The relatively small difference in overall survival indicates that a large portion of patients failing carboplatin single agent therapy can still achieve long-term cure with cisplatin-based salvage chemotherapy. Favourable results with salvage chemotherapy have also been reported for seminoma patients failing first-line cisplatin-based chemotherapy (Fléchon *et al*, 2001).

Based on the similar survival rates observed in both studies, we have combined the individual patient data from both the British and German randomised trials in order to definitively determine the role of carboplatin in a large group of seminoma patients as well as to investigate whether specific good prognostic patient subgroups with an equivalent outcome following either carboplatin single agent or cisplatin-based combination chemotherapy could be identified.

The results of our analysis clearly confirm the inferiority of carboplatin single agent treatment to cisplatin-based regimens regarding progression-free survival. The overall survival rate is also 5% lower following carboplatin therapy compared with cisplatin-based combinations. Even with the 361 patients from the two randomised trials, a clinically meaningful difference of 5% in overall survival could not be demonstrated with statistical significance, since this calculation would have needed more than 600 patients. However, with the consistency of the data reported in both trials, it is likely that a small but true survival difference exists in favour of cisplatin-based combinations.

It remains currently unclear whether carboplatin combination regimens may achieve an efficacy similar to BEP or VIP. Favorable results, comparable with the results achieved with carboplatin monotherapy in phase II studies, have been reported in phase II studies for the combination of carboplatin and cyclophosphamide or ifosfamide, but these regimens have never been tested within randomised phase III including only patients with advanced seminoma. The only three randomised studies published thus far comparing carboplatin with cisplatin-based combinations have mainly included patients with metastatic nonseminoma and only a small group of seminoma patients (Bajorin *et al*, 1993; Bokemeyer *et al*, 1996; Horwich *et al*, 1997).

As expected, cisplatin-based combination regimens were significantly more toxic than carboplatin single agent therapy. However, cisplatin-related toxicity was acceptable and did not outweigh the cisplatin-induced improvement in survival. According to the factors investigated by the IGCCCG study, we have differentiated six subgroups in our randomised patient population based on the presence of pulmonary metastases, presence of nonpulmonary visceral metastases or presence of lymph node metastases only in order to investigate the potential role of carboplatin single agent therapy among these separate patient cohorts with specific characteristics. However, no patient subgroup could be identified for which carboplatin had achieved an equivalent progression-free survival rate to cisplatin-based treatment. Even in patients with lymph node metastases only, a subgroup considered to exhibit a better prognosis than patients with visceral metastases, cisplatin-based combination therapy turned out to be significantly superior to carboplatin single agent therapy.

In conclusion, single agent carboplatin cannot be recommended as an equivalent therapeutic alternative to cisplatin-based combination therapy for patients with metastatic seminoma. Carboplatin may only be considered in those few patients who are unable to tolerate cisplatin-based regimens due to co-morbidities. Based on the results of the present analysis, these patients can now be exactly informed about their chances of cure and relapse rates when choosing carboplatin single agent treatment.
